# Application of McGuire’s Model to Weight Management Messages: Measuring Persuasion of Facebook Posts in the Healthy Body, Healthy U Trial for Young Adults Attending University in the United States

**DOI:** 10.3390/ijerph192114275

**Published:** 2022-11-01

**Authors:** Jeanie Arnold, Caitlin P. Bailey, W. Douglas Evans, Melissa A. Napolitano

**Affiliations:** 1 Department of Prevention and Community Health, Milken Institute School of Public Health, The George Washington University, Washington, DC 20052, USA; 2 Department of Exercise and Nutrition Sciences, Milken Institute School of Public Health, The George Washington University, Washington, DC 20052, USA

**Keywords:** McGuire’s Model, communication, Facebook, weight

## Abstract

Digital communication is a common intervention channel for weight loss, yet little is known about the types of messages that are most effective. Using McGuire’s Model of Communication and Persuasion as a framework, this study investigates the persuasiveness of Facebook messages posted as part of the weight loss intervention in the Healthy Body Healthy U (HBHU) study to determine what message characteristics prompt higher engagement on Facebook, and whether certain messages are more appealing to certain demographics. The first four weeks of HBHU Facebook posts (n = 32) were coded according to McGuire’s Input Communication Factors. Facebook engagement scores [(Total Engaged Users/Total Reach) × 100] were calculated for each post to determine effectiveness. The most effective posts were diet-related discussions or interactive polls. Participants who engaged with the highest and lowest effect posts were in their mid-twenties and tended to be female. Those engaged with the highest effect posts had an average BMI of 32.34 kg/m^2^, while those engaged with the lowest effect posts had an average BMI of 31.31 kg/m^2^. The least effective posts were didactic lessons (i.e., diet- or reminder-based), or video-based informational posts (edutainment). Future interventions should balance didactic content and interactive engagement to achieve persuasive messaging.

## 1. Introduction

The shift from adolescence to young adulthood is a pivotal and vulnerable one. Young adulthood, defined as ages 18–35, dovetails with increased obesity risk as well as excessive weight gain, a health problem that is associated with increased morbidity and mortality [1]. Over one third of young adults in the United States have obesity [2]. This population has an increased risk of cardiovascular disease, type 2 diabetes, obesity-related cancer, and mortality [3]. Despite the high risks associated with unhealthy weight in this developmental transition period, early adulthood is an often-neglected stage of life in the obesity literature [1].

The pervasiveness of social media and mobile technology for both information consumption and communication among young adults make it a sensible target platform for interventions. Cellphone ownership among young adults ages 18–29 in the United States is ubiquitous, and 96% own a smartphone [4]. Social media is a common form of communication for young adults and is often accessed on smartphones; 48% of young adults ages 18–29 in the United States say they are online “almost constantly” according to recent PEW data [5]. Among young adults ages 18 to 29 in the United States, 70% report using Facebook, establishing the platform as one of the most widely used despite the emergence of new platforms such as TikTok [6]. Systematic reviews of social media interventions for health behaviors, such as physical activity, show promise for outcomes including weight, physical activity and wellbeing [7,8].

Though social media is widely used by young adults, there are few weight loss interventions carried out through mobile platforms (including social media) that specifically target young adults. Literature reviews have found that among social media-delivered interventions in the United States, the operationalization of social media is varied, as is participant engagement [9]. Participant engagement is one of the indicators of weight loss success in these weight loss interventions; finding and establishing processes to deliver and measure engaging social media content is key to successful interventions [9,10]. Research also shows that early success in weight loss programs is indicative of continued weight loss success throughout the course of the program [11].

**Figure 1:**
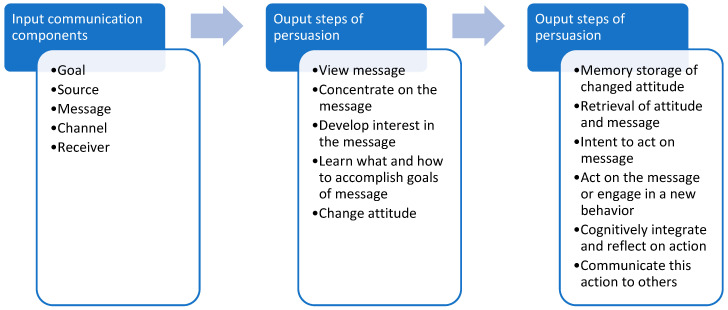
McGuire’s Communication and Persuasion Matrix (Adapted from McGuire, 2013).

The purpose of this study is an initial investigation of the persuasiveness of Facebook messages in weight loss interventions that target young adults using McGuire’s Model of Communication and Persuasion as a framework (see Figure 1). McGuire developed a matrix of variables meant to help define the mechanisms of persuasion in communication campaigns. Input Communication Factors pertain to the message being relayed to the audience and are manipulated by the author(s) of the message, while sequential Output Persuasion Steps are endpoints to determine the degree of success of the message and are related to the audience’s consumption of the message [12,13]. An effective message in an intervention or campaign can be a critical component in achieving behavior change, particularly if a program is delivered primarily online where messages must break through the noise of social media newsfeeds.

A literature search returned only a few examples of McGuire’s Model used as a framework in its totality. Bull et al. [14] used McGuire’s Model to characterize health education materials and test the impact of the various input characteristics on all ten outputs in the context of weight management behavior change. They reported that attractiveness, encouragement, level of information, application to one’s life, readiness to change, and self-efficacy were associated with McGuire’s output steps [14]. McGuire’s Model has also previously been used in a systematic review investigating Perceived Message Effectiveness (PME) in tobacco messaging [15]. Variables were adapted to form a rubric by which to categorize constructs and measure PME. This study used the Output Persuasion Steps only. Another study analyzing a communication campaign on body donation used Input Communication Factors for their analysis [16]. The use of Input Communication Factors was cited as a successful lens to understand the communication subject. Replicating the operationalization of Input Communication Factors is straightforward and systematic, thus this retrospective study will focus on those factors and how they relate to message interaction, an indicator of developing interest in a message (an Output Step). This is the first study to use McGuire’s Matrix to map out Facebook posts in a health intervention The objective of this analysis is to determine what message characteristics prompt higher engagement on Facebook, and to determine if certain messages are more appealing to certain demographics. The ultimate purpose of this inquiry is to inform intervention message creation for future digital-based health interventions.

## 2. Methods

### 2.1. Design and Sample

This analysis will use data from the Healthy Body Healthy U (HBHU) study conducted at the George Washington University (GWU) and the University of Massachusetts-Boston (UMB) in the United States [17]. This 18-month randomized, controlled clinical trial examined the efficacy of two social media-based interventions for weight loss among young adults ages 18–35 years attending a college or university in Washington, DC or Boston [18]. The two interventions were compared to a contact control group, and all were delivered via Facebook and SMS text messaging. Of the two interventions, one used Targeted (generic, appealing to a large audience) content and one used Tailored (personalized, relevant to an individual) feedback [17].

Study participants were recruited at GWU and UMB, though students in the eligible age range at any college or university in Washington, DC or Boston were eligible to take part. Other eligibility requirements included having a BMI of 25–45 kg/m^2^, English fluency, an active Facebook account, and regular text message access. Participants enrolled in cohorts from May 2015 to January 2018 and were randomized into one of the interventions or the control, resulting in 26 cohorts with participants in three different study groups in each cohort.

### 2.2. Selection Criteria

This retrospective observational analysis will use the intervention study groups (Tailored and Targeted in the HBHU study), excluding the contact control group, across all 26 cohorts. The contact control group will be excluded due to this analysis’ goal of focusing on weight loss intervention efficacy via Facebook. The Tailored and Targeted study arms will be included because their primary outcome is weight loss [17]. Study outcomes are reported elsewhere [18].

Study content delivery for the two weight loss focused treatments was through a combination of text messaging and Facebook posts. Facebook posts were centered on delivering the Diabetes Prevention Program [19] weight loss content through a combination of didactic and edutainment videos, as well as to prompt social support and engagement through polls and discussions. Between six and seven Facebook posts were delivered to cohort groups each week through Facebook groups during the 18-month intervention, resulting in over 400 delivered posts. This analysis will focus on the first four weeks of Facebook posts (n = 32) as engagement in the first four weeks is predictive of long-term success [11]. Week 1 delivered an additional introductory post, and there were six participant-generated posts in the first four weeks. The same Facebook posts were delivered on a consistent timeline to both the intervention arms of the study.

### 2.3. Framework

To investigate the persuasiveness of Facebook messages in this study, McGuire’s Model of Communication and Persuasion will be used as a framework [12,13]. An effective message in an intervention or campaign can be a critical component in achieving behavior change, particularly if a program is delivered primarily online. Persuasiveness is a complex construct. This retrospective observational analysis will adapt and apply McGuire’s input communication factors to analyze the first four weeks of Facebook posts delivered to the weight loss intervention groups to decipher commonalities across more engaging posts. McGuire’s original Communication-Persuasion Matrix can be seen in Figure 1.

By operationalizing McGuire’s Input Communication Factors in this context, characteristics of each post will be coded and examined for trends and patterns. Input Factors are manipulated by the program or intervention designer, while the Output Persuasion steps are participant endpoints that can be measured to determine the degree of success of the message. As an initial proof-of-concept, only Input Communication Factors will be examined among the Facebook posts.

### 2.4. Input Variables

In the McGuire framework, there are five elements (Source, Message, Channel, Receiver, Intent) that comprise Input variables (See Table 1). The traditional framework was adapted to better serve Facebook’s model of communication and the social media landscape. The adapted elements are described in detail below.

***Source***: There are three source designations possible in analyzing the study’s posts on Facebook. There is the “we” of HBHU and the study, coded as “HBHU-we”, for posts that present didactic or informative communications; there is the “I” of Georgie Beacon, coded as “HBHU-I”, for posts that present an “I” positioning (Georgie Beacon is the Facebook profile name representing HBHU in Facebook groups); and there are participants, coded as “Participant”, for posts that study participants publish.

**Figure 2:**
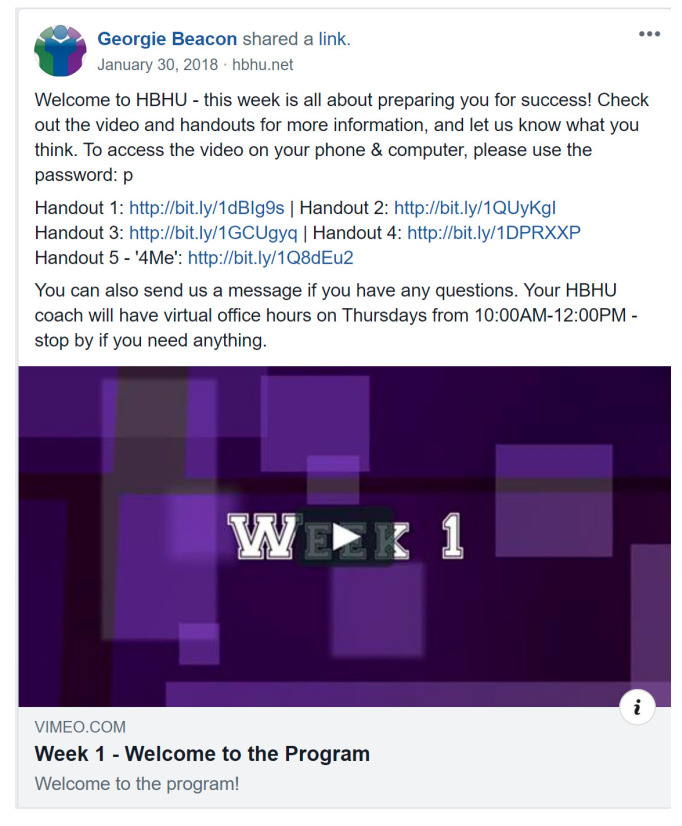
Sample weekly didactic lesson post by the study team (“Wk1_Tu” in Table 2).

**Figure 3:**
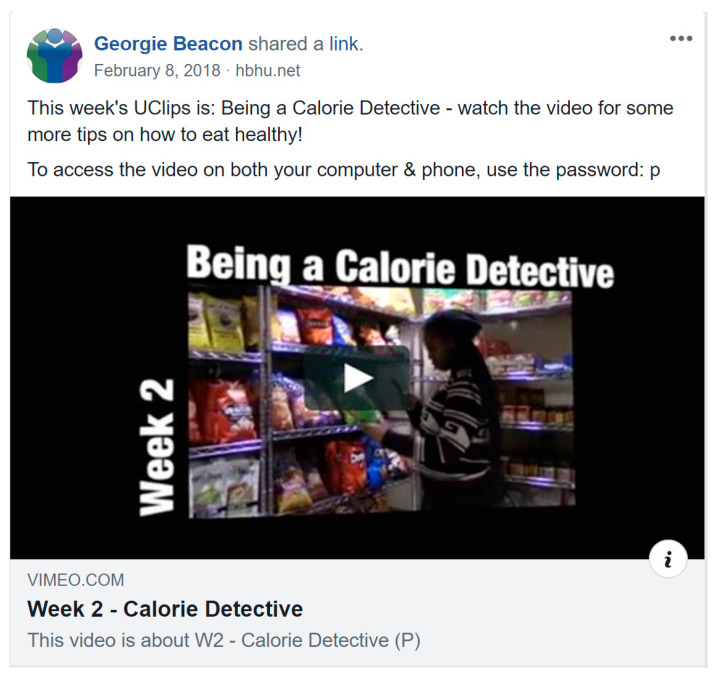
Sample weekly Didactic Vignette as posted by the study team (“Wk2_Th” in Table 2).

***Message***: There are several message designations in HBHU’s Facebook communications. “Didactic Lesson”, for weekly educational content from HBHU (see Figure 2); “Didactic Vignette”, for peer-modeled short form edutainment video content (See Figure 3); “Discussion”, for posts that seek to stir conversation; “Poll”, for posts that ask for a response to a poll; “Informational”, for posts that serve updates or administrative program information; and “Participant-Generated”, for posts created by study participants.

***Channel***: According to McGuire’s original framework, this *Channel* element would state HBHU’s posts were delivered through the channel of Facebook. Since all analyzed communications are specific to Facebook, *Channel* will instead be adapted to designate the post’s communication medium: paragraph (written text), picture, or video.

***Receiver***: This variable, meant to measure the audience of the message, has been divided into three demographic variables in order to quantify the profile of an engaged receiver. Demographic data for each study participant that engaged with a post was aggregated and an average was calculated for each post for the following demographic variables: age, sex, and BMI. Age and sex were collected from each participant at baseline. BMI was calculated from height and weight (kg/m^2^) measured at baseline. A BMI of 25–29.9 kg/m^2^ is in the overweight category. A BMI of 30 or greater is in the obesity category.

***Intent***: This is an adaptation of the traditional *Destination* variable. This indicates whether a post’s goal is for click-throughs (e.g., to click on a video or handout), reactions (e.g., to participate in a poll), or comments (e.g., to share personal stories or information).

***Theme***: This is an additional variable intended to categorize the various HBHU posts. There are three major themes: physical activity, diet, and lifestyle. This will help determine if some topics are more appealing or engaging to study participants than others. A “reminder” theme will also be included, for a total of four potential categories, to account for weekly administrative-style posts directing participants to check their emails or text messages.

***Effect***: Due to the available data, Facebook engagement scores will be used as a simplified output measure to determine degrees of success, or effectiveness (Effect). The traditional Facebook engagement equation of [(Total Engaged Users/Total Reach) x 100] will be calculated per post [20]. Engaged Users will be counted as study participants who engaged with a post (e.g., comment or like) and Total Reach will be counted as number of study participants who “Saw” the post. These scores were then classified into three categories: Low, Medium, and High.

### 2.5. Coding Plan

For this retrospective study, each post was coded for the five categorical variables listed above: Source, Message, Channel, Intent, and Theme. The Receiver variables (age, sex, BMI) were used to create an engaged participant demographic profile. For each post, the age, sex, and BMI of participants who engaged with the post (i.e., commented or ‘liked’) were aggregated to calculate an average “engaged participant demographic profile” for each post. For Sex, males were coded as 1 and females as 2. Effect scores were calculated using the aggregate engagement data and Facebook engagement equation described above. Of the Participant-Generated posts, one did not have available data to code for Channel, Theme, Intent, and Effect.

### 2.6. Analysis Plan

Post data was compiled in Microsoft Excel and sorted to visualize the data, and to characterize the Lowest and Highest Effect posts. Effect score (continuous variable) was transformed into a categorical variable (Low, Medium, High) using equal percentiles with two cutpoints.

Descriptive statistics for frequencies and means were run. Statistically significant differences between Effect score groups was tested using ANOVA tests. All statistical analyses were conducted using IBM SPSS (IBM, Armonk, NY, USA) for Windows.

## 3. Results

### 3.1. Facebook Post Overview

A total of 32 Facebook posts were coded for the six Input Variables (Source, Message, Channel, Receiver, Intent, Theme). A full list of post coding for all weeks can be found in Table 2. This includes Week 1 (9 posts), Week 2 (7 posts), Week 3 (9 posts), and Week 4 (7 posts). A summary of the highest and lowest effect posts can be found in Table 3.

### 3.2. Facebook Post Characteristics by Source

**Figure 4:**
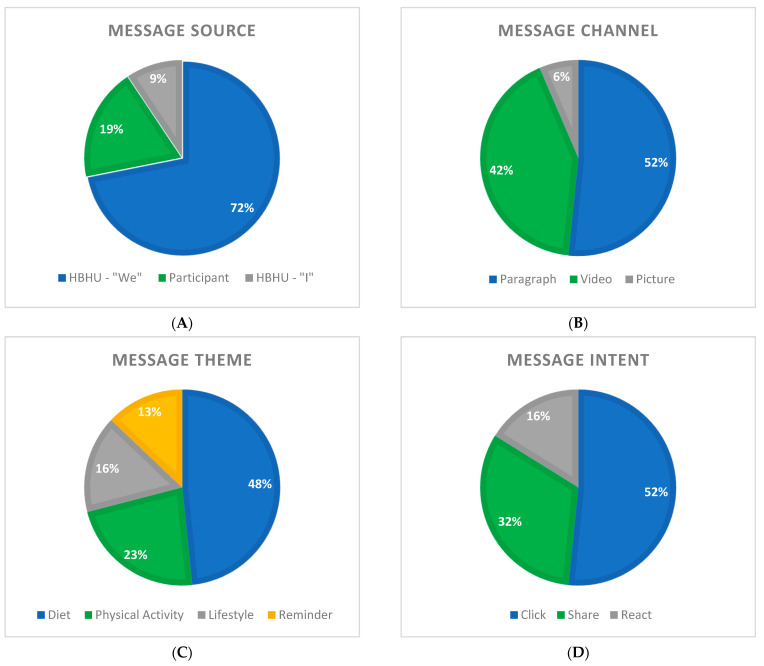

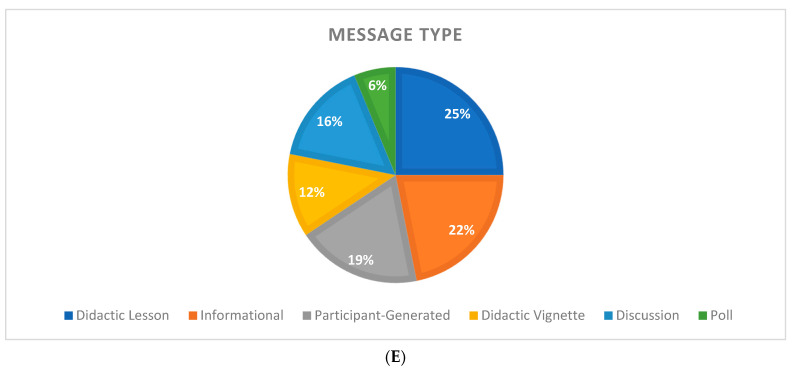
Frequencies of each coded variable across all Input Factors. (Panel **A**): Message Source (“we”, participant, “I”). (Pane **B**): Message Channel (paragraph, video, picture). (Panel **C**): Message Theme (diet, physical activity, lifestyle, reminder). (Panel **D**): Message Intent (click, share, react). (Panel **E**): Message Type (didactive lesson, informational, participant-generated, didactive vignette, discussion, poll).

Of the 32 posts, 23 were coded “HBHU-we”, three “HBHU-I”, and six were Participant posts (Figure 4A). All three “HBHU-I” posts were weekly wrap-up posts in Paragraph-form. These posts were meant to wrap up the previous day’s Discussions or Polls posed to participants. The Effect scores of these were split evenly across the High, Medium, and Low effect score categories, with the Week 2 Wrap Up scoring highest (Table 2).

“HBHU-we” posts were more varied across all other categories and functioned mostly as Didactic (n = 12) or Informational (n = 7) posts. Most were Videos (n = 13) or Paragraphs (n = 9). Diet was the most common Theme for the “HBHU-we” Source with ten posts, and only six “HBHU-we” posts scored High Effects while the other 17 were Medium or Low.

Participant-Generated posts were mostly in Paragraph-form with the Intent for other participants to Share (i.e., comment additional answers or information on the post). These posts had more High Effect scores but tended to be outliers: two had 100% and two 0%. The two 0% posts were both information-seeking questions directed at the study (e.g., “when is the best time to weigh in?”). The 100% score posts both invited discussion (e.g., sharing frustration about weight loss progress).

### 3.3. Facebook Post Characteristics by Channel

The majority of posts were delivered in Paragraph form (n = 16) followed by Video (n = 13) (Figure 4B). All Didactic Lessons and Vignettes were Videos. Most had a Medium Effect score (n = 6) and two scored a High Effect. Both High Effect videos were in Week 1: one was the overall Welcome post and the other was the first Didactic Lesson Video of the study.

### 3.4. Facebook Post Characteristics by Theme

Nearly half the posts focused on Diet, and another quarter on Physical Activity (Figure 4C). All Reminder posts were Informational with Low Effect scores. Of the Lifestyle-related posts, two were High Effect. These were the first two posts of the full HBHU study, both videos from “HBHU-we”. See Table 2.

Two Physical Activity posts were High Effect; one of those was the only Participant-Generated post that was not an outlier, with an Effect score of 33.33%. The other High Effect Physical Activity post was a Discussion post from “HBHU-we” that prompted participants to share their group fitness class preferences.

Six of the 15 Diet posts scored High Effects. Two of these were Participant-Generated outliers at 100% engagement. The other four were either Discussion or Poll posts prompting participants to Share. These four posts had the Highest Effect scores out of all coded posts that were not Participant-Generated.

### 3.5. Facebook Post Characteristics by Intent

A little over half the posts prompted participants to Click, 32% to Share (i.e., comment), and 16% to React (Figure 4D). All Didactic posts (both Lessons and Vignettes) were Click posts. These scored mostly Low and Medium effect scores except for one Didactic Lesson post; this post was the very first Lesson post for the full HBHU study (Table 2).

All React posts were Informational posts from “HBHU-we” and scored Medium and Low effects.

After removing the four outlier Participant-Generated posts from the Share posts, five of the six posts scored High effects. These were either Polls or Discussions, and all but one was Diet-related. All were either a Wednesday post from “HBHU-we” or a Wrap Up post from “HBHU-I.”

### 3.6. Facebook Post Characteristics by Message Type

Most of the Message types were Didactic Lessons (25%), Informational (21.9%), or Participant-Generated (18.8%) (Figure 4E).

Didactic Lesson posts (n = 8) were all coded as “HBHU-we”, and all were Videos. See Table 2. There was nearly an even split on Theme, with three Diet, three Lifestyle, and two Physical Activity. All were coded with Click Intents, and only one resulted in a High Effect. These posts were all either a Tuesday or Friday post with the lesson for the week and handouts.

Didactic Vignettes (n = 4) were all “HBHU-we” and appeared in Video form. The Theme was primarily Diet, with Click Intent. Most of the Vignette posts had a Medium Effect. These posts all happened on Thursdays and were short videos meant to reinforce the weekly lessons.

Discussion posts (n = 5) were split between HBHU “we” and “I”, and all were Paragraphs. Themes were evenly split between Diet and Physical Activity. Three prompted a Share, and three achieved a High Effect.

Informational posts (n = 7) were all from “HBHU-we”. The majority were Paragraphs, and most were Reminders. Five of the posts prompted Reacts as their Intent, and only one achieved a High Effect while the others were evenly split between Medium and Low.

Poll posts (n = 2) were both from “HBHU-we”, and both in Paragraph form. They related to Diet and prompted participants to Share; both achieved a High Effect score. The two Poll posts were in the top three Highest Effect scoring posts that were not Participant-Generated.

Of the Participant-Generated posts (n = 6), only five had sufficient recorded data to code for all input variables. Most were in Paragraph form and related to either Diet or Physical Activity. Their Intent was primarily Sharing, and their Effects were split between High and Low. As stated earlier, many of these are outliers.

### 3.7. Facebook Post Characteristics by Receiver

Demographic data for each participant who engaged with each post was aggregated and averages were calculated for each variable on each post to create a “Receiver Profile” of who was interacting with each post (Table 2). Overall, engaged participants were more likely to be female, in their mid-twenties, with a BMI in the obesity range. The Receiver age ranged from 20 to 29 years, with an average 24.32 ± 1.56 years; the Receiver sex ranged from 2 to 1.67; and the Receiver BMI ranged from 27.08 to 34.11 kg/m^2^, with an average BMI of 31.54 ± 1.42 kg/m^2^.

### 3.8. Facebook Posts Characteristics by Effect

**Figure 5:**
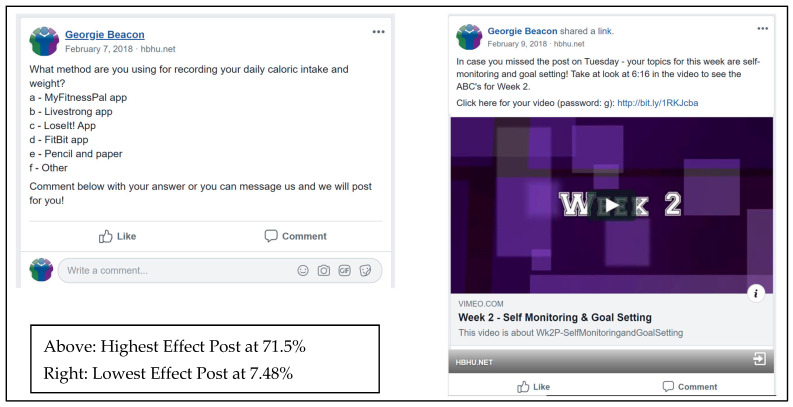
Highest vs. Lowest Effect Post as they appeared on Facebook. Note: For coding data, see Wk2_Wednesday for Highest and Wk2_Friday for lowest in Table 2.

Post effect scores ranged from 0 to 100% before eliminating the Participant-Generated Outlier posts. Without outliers, the Highest Effect post (Wk2_W in Table 2) scored a 71.50% effect, while the Lowest Effect post (Wk2_F in Table 2) scored a 7.48% (Figure 5). After using IBM SPSS to bin post Effect scores into three categories, there were eleven Low Effect (0–11.76%), ten Medium Effect (11.76–20.24%), and ten High Effect (20.24–100%). Removing the four outlier posts resulted in eight High Effect Posts and nine Low Effect Posts. A summary of the Highest and Lowest Effect Posts can be found in Table 3.

Six of the eight High Effect posts were coded for “HBHU-we” as their source. Three were Discussion messages, with two more Poll messages, meaning all Polls were High Effect. Five were in Paragraph-form, and four were Diet-themed. Five were coded as a Share Intent.

Eight of the nine Low Effect posts were also coded as “HBHU-we”. The most common Message code was Didactic Lesson (four of nine), followed by Informational (three of nine). Didactic Lessons and Informational posts were the most common of all Message types in the overall coding. It should be noted that the bottom three Didactic Lesson posts were all lesson recap-style posts; they served to reiterate the lesson from earlier in the week for the HBHU study. Five of the nine were Videos, four of the nine were Diet, followed by three Reminders. Six of the nine posts were coded as Click for Intent.

In summary, the average High Effect post in this study came from “HBHU-we”, was a Discussion or Poll in Paragraph-form, focused on Diet, and prompted participants to Share. The average Effect score for High Effect posts was a 34.08%. The average Low Effect post in this study came from “HBHU-we”, was a Didactic Lesson or Informational post in Video-form, focused on Diet or Reminders, and prompted participants to Click the post. The average Effect score for Low Effect posts was 10.37%. A summary can be found in Table 3.

### 3.9. Participant Demographics and Message Effects

No statistically significant differences were found between Effect categories for Age, Sex, or BMI. A summary of Age, Sex, and BMI for each Effect category (Low, Medium, High) can be found in Table 2.

## 4. Discussion

The purpose of coding and analyzing these Facebook posts was to explore characteristics of effective messages and what was most effective, including learning if certain messages were more appealing to some demographics rather than others. This is the first time McGuire’s Matrix has been used to map out Facebook posts in a health intervention. Practitioners and future researchers can use these findings to develop health messaging that is engaging to young adults, ultimately making messages that are more likely to impact behavior, per McGuire’s Model.

After comparing the posts in the High and Low Effect categories, findings indicate Polls and Discussion posts were more effective and engaging than Didactic Lessons and Informational Reminder posts. Participant-Generated posts also tended to be either very High (100%) or very Low (0%) in Effect. However, those posts only appear to the cohort page they posted in, meaning there is a much smaller pool of participants who can see and engage with each post. Because of this, many of these Participant-Generated posts are likely to be outlier posts and have been excluded from aggregate comparisons. Focusing specifically on Participant-Generated posts and the profiles of those that create and engage with them would be an interesting avenue for future research.

Inviting audiences to be active participants yields higher engagement. Finding Polls and Share Intents to be most engaging is consistent with a previous study’s conclusion that Facebook posts that seek feedback and audience participation, such as polls, are more engaging than other post types in a Facebook-based weight loss intervention [10]. As “HBHU-we” and Diet were the most frequent codes for Source and Theme, further post coding would need to be done before assigning any significance to those results or categories.

The high effectiveness of Paragraph-form posts and relatively low engaging Video-form posts contradicts Facebook marketing best practices. Standard Facebook engagement advice, starting in 2016, dictates the use of pictures whenever possible and to take advantage of video, as video is high performing and highly engaging [21]. This is an important finding, and additional coding should be done to see if this continues to be the case for Videos and Paragraphs in the context of Facebook weight loss interventions. Other emerging research has also found that, in certain advertisement contexts, images are more appealing than videos to users [22]. It is logical to look to existing advice on social media engagement as health interventions dip into these platforms, but prudent to ensure those commercial marketing best practices align with the social marketing context for these interventions.

No significant differences were found between Low and High Effect groups for any of the demographic variables, however BMI was approaching significance and additional coding might reveal significance for that variable. Previous studies have found social media engagement to be related to weight loss in the context of an intervention, and knowing what participants are more likely to engage with social media posts would prove useful in targeting content [10].

### Limitations

The HBHU study began in 2015 and finished in 2019. Facebook is known for constantly changing its Newsfeed algorithm, which determines how posts (ads, “Friend” posts, or posts from groups a user belongs to) appear on the user’s Newsfeed, including a 2018 update prioritizing content from “Friends” [23]. With cohorts enrolling at different times, it is possible study participants experienced HBHU posts under different algorithm environments, which would impact engagement. We purposely selected Facebook for our study, as it had the ability to create online groups and we could post a large amount of content. However, this is one of the limitations of the application of McGuire’s framework as the study was only investigating posts and engagement from a reputable source. Future studies might investigate the use of other, newer social media platforms (e.g., Tiktok), for health messaging dissemination, as well as implications for the engagement with mis- and disinformation [24,25]. Such work could be particularly useful regarding matching channel to delivery purpose. For example, Tiktok, a platform designed for posting and viewing video content, may be more effective for disseminating video lessons compared with Facebook.

Cohort size for the HBHU study ranged from 7 to 30 participants for the UMB groups (an average of 18.75 participants per cohort), and 7 to 22 for the GWU groups (an average of 16.79 participants per cohort). The range in sizes in each Facebook group also has the potential to impact engagement scores.

For this analysis, only the first four weeks of posts were coded and analyzed. It should be noted much of the first four weeks focused on diet (Figure 4C). Future research should expand to a larger selection of data to achieve a clearer and more complete idea of how Facebook post characteristics and engagement are related. This, however, proves to be a promising start. This is also a slightly incomplete data picture in that clickthrough data for the video links was unavailable, as this study was not designed for this retrospective analysis. Clickthrough data could change how engaging some of the lower effect posts were, and future interventions aiming to measure constructs like engagement at a more granular level should take this into consideration.

## 5. Conclusions

In this study, we found that Polls and Discussion posts were more effective and engaging than Didactic Lessons and Informational Reminder posts. Systematically categorizing and quantifying Communication Input Factors with McGuire’s Matrix [12,13] as a framework has several applications, including the design and evaluation of future health interventions and other social marketing campaigns. Establishing a consistent framework by which to design content in the constantly evolving world of social media is important as social media interventions continue to gain popularity and prove useful. If we cannot properly evaluate their success or processes, we cannot replicate that success with any degree of certainty.

Coding posts beyond the first four weeks of the HBHU study should be pursued to determine if Paragraph-form posts were indeed more engaging than Video-form posts. As stated before, this contradicts current Facebook best practices [21]. This would present an interesting quandary for intervention designers, as weight loss interventions delivered via social media are still newer to the scientific literature: does social media engagement differ in an intervention context versus regular use? That is, can intervention designers confidently look to existing commercial marketing success strategies for social marketing as they have for other mediums, or will social media use a different set of rules?

The high engagement Effect of posts that prompt a Share response aligns with previous research and should be kept in mind as researchers move forward with social media interventions [10]. Polls have proved to be engaging and this format should be leveraged for future interventions. Using Polls as a teaching tool in addition to an engagement tool should be tested in conjunction with more traditional Didactic Lesson media.

Social media is a relatively inexpensive intervention platform used by many diverse groups. Researchers are finding more ways to leverage it for interventions; however, social media is constantly updating and changing. Creating ways to create and deliver engaging content as well as effectively measure the processes and outcomes is crucial to success. McGuire’s Input Factors have the potential to be used systematically across social media content creation and analysis, as demonstrated by this retrospective study, and can be used in conjunction with social media’s traditional engagement equations. This is a promising avenue for future evaluation and design as social media interventions become more widespread.

## Figures and Tables

**Table 1:** Adaptation of McGuire’s Model of Communication and Persuasion [13].

Input Factor	Definition	Adaptation
Source	Who is delivering the message?	Facebook posts in cohort groups could come from one of two sources: the HBHU study, or participants. HBHU posts were divided in two based on if the post language used “we” or “I” as the speaker.
Message	What form does the message take?	HBHU’s Facebook posts: “Didactic Lesson”; “Didactic Vignette”; “Discussion”; “Poll”; “Participant-Generated”.
Channel	How is the message delivered?	Designates the post’s communication medium: paragraph (written text), picture, or video
Theme	What is the theme of the message?	This is an additional variable intended to categorize the various HBHU posts. There are three major themes: physical activity, diet, and lifestyle.
Intent	What action does the message call for?	This represents the goal of the post: for click-throughs (e.g., to click on a video or handout), reactions (e.g., to participate in a poll), or comments (e.g., to share personal stories or information)
Receiver	Who is the message directed at?	To measure the audience of the message, this variable has been divided into three demographic variables (Age, Sex, BMI) in order to quantify the profile of an engaged receiver.
Effect	How successful is the message?	Facebook engagement scores will be used as a simplified output measure to determine degrees of success, or effectiveness (Total Engaged Users/Total Reach).

**Table 2:** Coded date for all posts (n = 32).

Week_Post	Source	Message	Channel	Theme	Intent	Receiver Age	Receiver Sex	Receiver BMI	Effect Score	Effect %
Wk1_Participant	Participant	Participant-Generated	Paragraph	Diet	Share	20.00	2.00	30.60	1.00	100.00
Wk3_Participant	Participant	Participant-Generated	Paragraph	Diet	Share	24.00	1.67	32.76	1.00	100.00
Wk2_Wednesday	HBHU	Poll	Paragraph	Diet	Share	23.79	1.86	31.53	0.71	71.50
Wk1_Wednesday	HBHU	Discussion	Paragraph	Diet	Share	24.64	1.90	32.36	0.35	35.29
Wk4_Wednesday	HBHU	Poll	Paragraph	Diet	Share	24.40	1.84	31.88	0.34	33.53
Wk1_Participant2	Participant	Participant-Generated	Picture	Physical Activity	Click	26.50	2.00	32.16	0.33	33.33
Wk2_WrapUp	Georgie	Discussion	Paragraph	Diet	Share	23.66	1.97	31.91	0.29	28.79
Wk3_Wednesday	HBHU	Discussion	Paragraph	Physical Activity	Share	24.42	1.91	31.56	0.28	27.56
Wk1_Tuesday	HBHU	Didactic Lesson	Video	Lifestyle	Click	24.61	1.87	34.07	0.22	22.16
Wk1_Welcome	HBHU	Informational	Video	Lifestyle	Click	24.63	1.94	33.25	0.20	20.47
Wk1_Thursday	HBHU	Didactic Vignette	Video	Lifestyle	Click	24.44	1.88	31.77	0.20	20.24
Wk3_environment	HBHU	Informational	Picture	Diet	Click	23.70	1.85	30.85	0.20	20.00
Wk1_WrapUp	Georgie	Discussion	Paragraph	Diet	Share	23.30	1.91	32.93	0.19	18.70
Wk3_Thursday	HBHU	Didactic Vignette	Video	Physical Activity	Click	23.25	1.88	31.13	0.17	16.55
Wk2_Monday	HBHU	Informational	Paragraph	Reminder	React	24.45	1.82	32.57	0.16	15.60
Wk2_Tuesday	HBHU	Didactic Lesson	Video	Lifestyle	Click	22.82	1.77	32.47	0.15	14.77
Wk2_Thursday	HBHU	Didactic Vignette	Video	Diet	Click	23.16	1.68	30.80	0.14	14.49
Wk3_Tuesday	HBHU	Didactic Lesson	Video	Physical Activity	Click	24.80	1.85	31.42	0.14	13.79
Wk4_WrapUp	HBHU	Informational	Paragraph	Diet	React	24.83	1.92	30.40	0.12	12.37
Wk3_Friday	HBHU	Didactic Lesson	Video	Physical Activity	Click	25.07	1.79	32.54	0.12	12.17
Wk4_Thursday	HBHU	Didactic Vignette	Video	Diet	Click	24.50	1.88	31.54	0.12	11.76
Wk4_Monday	HBHU	Informational	Paragraph	Reminder	React	24.60	1.80	32.48	0.12	11.63
Wk3_Monday	HBHU	Informational	Paragraph	Reminder	React	23.65	1.82	31.26	0.12	11.56
Wk4_Tuesday	HBHU	Didactic Lesson	Video	Diet	Click	23.80	1.87	31.41	0.11	11.28
Wk1_Monday	HBHU	Informational	Paragraph	Reminder	React	24.33	1.72	34.11	0.11	11.11
Wk3_WrapUp	Georgie	Discussion	Paragraph	Physical Activity	Click	25.60	2.00	30.28	0.11	10.71
Wk1_Friday	HBHU	Didactic Lesson	Video	Lifestyle	Click	25.78	2.00	29.18	0.10	9.52
Wk4_Friday	HBHU	Didactic Lesson	Video	Diet	Click	23.36	1.73	31.44	0.08	8.27
Wk2_Friday	HBHU	Didactic Lesson	Video	Diet	Click	21.73	1.73	30.04	0.07	7.48
Wk2_Participant	Participant	Participant-Generated	Paragraph	Diet	Share	29.00	2.00	29.82	0.00	0.00
Wk4_Participant	Participant	Participant-Generated	Paragraph	Physical Activity	Share	27.00	2.00	27.08	0.00	0.00
Wk3_Participant2	Participant	Participant-Generated	N/A	N/A	N/A	N/A	N/A	N/A	N/A	N/A

Note: Participant-Generated posts were limited to the cohort page they were posted on, and are greyed out as outliers. Posts are sorted by % Effect (highest to lowest).

**Table 3:** Summary of Highest vs. Lowest Effect Posts.

**Highest Effect Posts**	**Source**	**Message**	**Channel**	**Theme**	**Intent**	**Age**	**Sex**	**BMI**	**Effect %**
**In general**	HBHU-we	Discussion OR Poll	Paragraph	Diet	Share	24.58	1.91	32.34	34.08
**Lowest Effect Posts**	**Source**	**Message**	**Channel**	**Theme**	**Intent**	**Age**	**Sex**	**BMI**	**Effect %**
**In general**	HBHU-we	Didactic Lesson OR Informational	Video	Diet OR Reminder	Click	24.15	1.84	31.31	10.37
